# Effect of prolonged intravenous glucose and essential amino acid infusion on nitrogen balance, muscle protein degradation and ubiquitin-conjugating enzyme gene expression in calves

**DOI:** 10.1186/1743-7075-5-5

**Published:** 2008-02-12

**Authors:** Fouzia Sadiq, Leslie A Crompton, Jes R Scaife, Michael A Lomax

**Affiliations:** 1 Hepatology Section, Imperial College, London W2 1PG, UK; 2 Department of Agriculture, University of Reading, Reading, RG6 6AR, UK; 3 Centre for Equine and Animal Science, Writtle College, CM1 3RR, UK; 4 Division of Nutritional Sciences, University of Nottingham, Sutton Bonington Campus, LE12 5RD, UK

## Abstract

**Background:**

Intravenous infusions of glucose and amino acids increase both nitrogen balance and muscle accretion. We hypothesised that co-infusion of glucose (to stimulate insulin) and essential amino acids (EAA) would act additively to improve nitrogen balance by decreasing muscle protein degradation in association with alterations in muscle expression of components of the ubiquitin-proteasome proteolytic pathway.

**Methods:**

We examined the effect of a 5 day intravenous infusions of saline, glucose, EAA and glucose + EAA, on urinary nitrogen excretion and muscle protein degradation. We carried out the study in 6 restrained calves since ruminants offer the advantage that muscle protein degradation can be assessed by excretion of 3 methyl-histidine and multiple muscle biopsies can be taken from the same animal. On the final day of infusion blood samples were taken for hormone and metabolite measurement and muscle biopsies for expression of ubiquitin, the 14-kDa E2 ubiquitin conjugating enzyme, and proteasome sub-units C2 and C8.

**Results:**

On day 5 of glucose infusion, plasma glucose, insulin and IGF-1 concentrations were increased while urea nitrogen excretion and myofibrillar protein degradation was decreased. Co-infusion of glucose + EAA prevented the loss of urinary nitrogen observed with EAA infusions alone and enhanced the increase in plasma IGF-1 concentration but there was no synergistic effect of glucose + EAA on the decrease in myofibrillar protein degradation. Muscle mRNA expression of the ubiquitin conjugating enzyme, 14-kDa E2 and proteasome sub-unit C2 were significantly decreased, after glucose but not amino acid infusions, and there was no further response to the combined infusions of glucose + EAA.

**Conclusion:**

Prolonged glucose infusion decreases myofibrillar protein degradation, prevents the excretion of infused EAA, and acts additively with EAA to increase plasma IGF-1 and improve net nitrogen balance. There was no evidence of synergistic effects between glucose + EAA infusion on muscle protein degradation or expression of components of the ubiquitin-proteasome proteolytic pathway.

## Background

The relative rates of muscle protein synthesis and degradation are critical for maintaining lean tissue mass and normal healthy metabolic function [1]. Using tracer incorporation into muscle, leg amino acid exchange and whole body measurements, it has been shown that the combined infusion or ingestion of carbohydrate and protein/amino acids are needed to increase whole body protein synthesis rate, to reduce protein degradation, and thus to elicit a positive net protein balance under resting conditions [2-5]. In a number of clinical conditions (e.g. postoperative-surgical trauma, sepsis, starvation) muscle wasting and negative net protein balance can be prevented by parenteral or intravenous infusions of glucose and amino acids but the optimum nutrient infusion mixture and mechanisms involved, remain to be defined [6].

Acute (3 h) infusions of insulin and BCAA, act synergistically to increase muscle protein synthesis [7], but there have been few studies on responses in muscle protein degradation, particularly over chronic periods of several days. Our previous studies *in vitro *have demonstrated an additive inhibitory action of insulin and amino acids on protein degradation in C2C12 myotubes [8]. Studies in animals and humans *in vivo *have failed to support a synergistic role for acute (3 h) infusions of glucose and amino acids in muscle protein degradation [7,9,10]. It has been proposed that insulin and amino acids may influence muscle protein accretion and degradation only during prolonged infusions [9].

The ubiquitin 26S proteasome dependent system is the predominant cellular mechanism for myofibrillar protein degradation in skeletal muscle [11,12]. Starvation up-regulates ubiquitin-dependent-proteolysis in muscle and this can be reversed on refeeding and insulin treatment [9,13] suggesting increased availability of amino acids and anabolic hormones may play a vital role in the down regulation of ubiquitin proteasome pathway. Targetting of myofibrillar proteins to the proteasome complex requires the addition of polyubiquitin chains to the protein substrate. Three enzymes are thought to regulate this process: ubiquitin activating enzyme E1, ubiquitin-conjugating enzyme, 14-kDa E2 and ubiquitin-protein ligase E3. Expression of ubiquitin and 26S proteasome subunits are thought to adapt to changes in flux through the pathway rather than being regulatory [1]. Our previous studies in C2C12 myotubes have implicated the ubiquitin proteasome pathway in the additive effect of insulin and amino acids on myofibrillar protein degradation [8].

We have employed ruminants to examine the effect of vascular infusions of insulin and branch chain amino acids on muscle metabolism *in vivo *[14,15]. Ruminants offer several advantages over rodents for the study of mechanisms regulating protein metabolism: foregut fermentation of carbohydrate results in less episodic changes in plasma glucose and insulin; prolonged infusions of glucose do not produce insulin resistance as reported in rats [16] due to the reduced expression of Glut 4 in muscle [17]; the rate of myofibrillar protein degradation can be assessed from 3-MH excretion. Additionally it is possible to maintain animals on restricted diets in an inactive state for long periods and take multiple muscle biopsies from the same animal.

We therefore conducted an *in vivo *study to examine the hypothesis that co-infusion of glucose and EAA would act synergistically to improve nitrogen balance by decreasing muscle protein degradation in association with alterations in muscle expression of components of the ubiquitin-proteasome proteolytic pathway. We infused EAA since this provided the limiting amino acids for protein deposition and BCAA signals without NEAA which are normally deaminated to supply carbons for gluconeogenesis. We show that prolonged infusion of glucose and EEA act additively to improve nitrogen balance but that EAA do not influence the inhibitory effect of glucose on muscle protein degradation and mRNA levels for the ubiquitin conjugating enzyme, 14-kDa E2 and proteasome sub-unit C2.

## Methods

### Experimental animals

All experimental procedures were conducted in accord with the principles of the UK Animals (Scientific Procedures) Act 1986. Six *Holstein-Friesian *male calves, 4 months of age and weighing 90 ± 1.9 kg were housed separately in metabolism stalls. Calves were fed a diet of weaner pellets and hay, (2:1) to restrict their growth rate to 0.3 kg/day, approximately one third of their growth potential. Over the experimental period calves were fed an average of 18 MJoules metabolisable energy and 36 g nitrogen per day. They were fed twice every day at 8:00 and 16:00 hrs and were given free access to water.

### Infusions

Once calves were adapted (1 month) to the diet, they were allocated at random to the following infusion treatments: Control (saline), Glucose, EAA, Glucose + EAA. Infusion treatments were infused continuously for 5 days via jugular vein catheters using an automated peristaltic pump (Gilson Minipuls (MP4), Anachem Ltd, Bedfordshire, UK). Catheters were inserted into both jugular veins under local anaesthesia, one day prior to the start of the infusion period. One catheter was used for infusion and the other for blood sampling. Weights of the infusates were recorded prior to onset of infusions and at least thrice a day thereafter to adjust infusion rates, if necessary. Catheters were monitored for blockage or infection and were flushed through with heparinized saline (50 IU/ml) twice a day.

Calves were infused with two different glucose infusion rates; 20 μmol glucose/kg BW/min and 9.6 μmol glucose/kg BW/min equivalent to 21.6 and 10.4 kCal/kg/day, respectively, such that six infusions (control, glucose × 2, EAA, glucose × 2 + EAA) were given in a 3 × 2 factorial design over a period of 8 weeks. Although the two glucose infusion rates gave dose dependent increases in plasma glucose and insulin, there were no differences between glucose infusion rates in the effects on protein metabolism so results for the two rates have been combined. The average of the glucose infusion was equivalent to 37% of metabolisable energy intake. Glucose (Sigma) infusate was sterilised through 0.45 μm filter and then autoclaved for 20 min at 15 psi Hg mm/120°C.

The mixture of EAA (Forum Pharmaceuticals & Fine Chemicals, Surrey) was infused at 0.8 mg/kg BW/min; this is approximately 50% of the EAA absorption rates into the portal vein observed for a 90 kg calf [18]. The mixture of individual EAA was made to achieve the following infusion rates (mg/kg/day) based on the composition observed in the portal vein of calves [18,19]; isoleucine, 0.117; leucine, 0.159; threonine, 0.085; valine, 0.137; methionine, 0.056; tyrosine, 0.118; phenylalanine, 012; tryptophan, 0.023; lysine, 0.165; histidine, 0.047; arginine, 0.126. The EAA were dissolved in 1.5 litre of ultra pure water and infused at a rate of approximately 1 ml/min, with the rates adjusted for the differences in the weight of the calves. Amino acid infusates were filtered sterile through 0.2 μm filters.

### Sampling

On the fifth day of each infusion period, blood samples (10 ml) were collected every 15 min during the last 6 hours of the infusion, using heparinized syringes. Plasma was separated immediately and kept at -20°C pending analysis. Muscle biopsy samples from the hind limbs were collected under local anaesthesia after the last blood sample was taken. Biopsy samples (approx 0.5 gm) were taken from the *biceps femoris*, carefully avoiding previous biopsy sites, taken for the other infusion treatments, and snap frozen in liquid nitrogen and transferred to -80°C freezer until required. The total urine output of each animal was collected daily into plastic containers containing 100 ml 6 N HCl during the last four days of the infusion period and a % 5 sample of urine was kept at -20°C until further analysis.

### Plasma metabolite, hormone and amino acid analysis

Plasma glucose and urea nitrogen levels were measured by the Trinder method and plasma urea N endpoint, respectively (SIGMA, Poole, Dorset, United Kingdom). Plasma insulin, IGF-1 and GH concentrations were measured by radio immunoassay using double antibody method [20]. Plasma amino acid concentrations were measured by ion exchange chromatography with ninhydrin detection using a Biochrom 20 amino acid analyser (Biochrom, Cambridge). Measurement of plasma metabolite and hormones were made on 6 samples taken at hourly intervals from each animal, except for GH, where samples were collected every 15 min during the last 6 hours of the infusion, and amino acids where a pooled sample representative of the 6 hour sampling period, was analyzed. Plasma 3-MH was measured as described for urinary 3-MH.

### Urinary metabolite analysis

Urinary creatinine and urea nitrogen levels were assayed colorimetrically using SIGMA kits, procedure nos. 555 and 640, respectively. Urinary 3-MH concentration was measured by HPLC as described by [21]. Urine samples were deproteinised and derivatised with fluorescamine (SIGMA F-9015) prior to measurement. Fluorescamine derivatives of 3-MH are retained on a C18 reverse phase column and can be eluted using an acetonitrile mobile phase.

A calibration curve was produced for 3-MH (SIGMA M-3879) using a set of standards (0.20, 0.15, 0.10, 0.05 and 0.025 mM). Two hundred micro litres of 0.2 M histidinol (internal standard; SIGMA H-6647) was pipetted in to 200 μl of samples and standards. Samples and standards were then deproteinized by adding 200 μl of 3 M perchloric acid followed by centrifugation at 2,300 g for 15 minutes at 4°C. Next 400 μl of double distilled H_2_O, 40 μl of 1.5 M NaOH and 400 μl of 0.2 M Na_2_B_4_O_7_, pH 9.0 were added into 100 μl of supernatants. Tubes were vortexed and derivatized with 0.25 ml of fluorescamine (0.16 g/100 ml acetonitrile). After 30 seconds 400 μl of 2 M HCl was added in all of the tubes to produce the stable form of 3-MH derivative. Tubes were vortexed again, capped and incubated at 90°C in water bath for 45 minutes. Samples/standards were extracted twice with 1.5 ml diethyl ether (HPLC grade) and bottom aqueous layer was transferred to HPLC vials (Thomson scientific, Cults, Aberdeen).

Seventy microlitre samples/standards were eluted through ODS (C – 18 reverse phase) column (Capital HPLC Ltd, Broxburn, West Lothian U. K.) using binary gradients. Ammonium acetate (8 mM) and acetonitrile (HPLC grade) were used as solvent A and B, respectively. The gradient rise was from 0.35 to 0.70 acetonitrile over 11 minutes at solvent flow rate of 1.1 ml/minute. Peaks were detected at 470 nm emission and 380 nm excitation.

### Northern blot analysis

Total RNA from muscle biopsy samples was extracted by the guanidine isothiocyanate method [22]. The ratio of A_260_/A_280 _was approx 2.0, indicating purity of total RNA preparations. Ten micrograms of RNA was run on 1% agarose formaldehyde denaturing gel, transferred to Genescreen™ membrane (NEN Life science products, Inc. Boston, Massachusetts, USA) and covalently fixed to the membrane by UV irradiation. The strippable cDNA probes, for 14-kDa E2 ubiquitin conjugating enzyme, C2 and C8 26S proteasome subunits, polyubiquitin and GAPDH, (a house keeping gene) were synthesized with [α-^32^P] dATP to a specific activity of 10^9 ^cpm/μg cDNA using Ambion Strip EZ DNA kit (AMS Biotechnology Ltd, Witney Oxon, UK). Unincorporated radioactivity was removed by passing the synthesized probe through chromaspin-30 column (Clonetech laboratories Ltd, Hampshire, UK). Membranes were prehybridised with Quickhyb hybridisation solution (Stratagene cloning system, California, USA) for 20 min at 65°C. Purified probe containing salmon sperm DNA (Ambion) was denatured at 90°C for 10 min and transferred to the hybridisation solution. Probe was then hybridised to the RNA on the blot for two hours at 65°C. To remove non-specific binding the blot was washed twice with 2 × SSC/0.1% SDS at 60°C for 15 min each and then once with 1 × SSC/0.1% SDS at 65°C for 15 min. Autoradiographic signals were quantified by laser densitometry and expressed relative to the corresponding GAPDH mRNA value to normalise for equal loading and transfer of RNA. GAPDH was used because previous studies with nutrients and hormones have documented that mRNA level for it remains constant [23]. Membranes were stripped using Strip EZ kit and rehybridised with other components of the ubiquitin proteasome pathway as described above. Autoradiographic signals for the 1.2 and 2.6 kb transcripts for 14-kDa E2 and polyubiquitin, respectively were normalised for GAPDH expression and presented as arbitrary values.

### Calculations and statistics

Myofibrillar protein degradation was estimated by expressing 3-MH urinary excretion rates relative to urinary creatinine excretion to compensate for differences in the muscle 3-MH pool size. Net nitrogen balance is the difference between digested nitrogen intake (assuming a nitrogen digestibility of 0.85) and urinary nitrogen excretion. Results from the two glucose infusion rates were pooled since there were no differences in the effects on protein metabolism. Differences of means were determined by two-way analysis of variance using general linear model (Minitab version 13.1, Pennsylvania, PA, USA). Post hoc comparisons were performed by Tukey's pair wise comparison for multiple treatments. A regression analysis was conducted to determine any correlation between parameters.

## Results

Calves were fed a diet to restrict growth rate to increase the sensitivity of muscle protein degradation to the infusion treatments. Our previous study [15] has demonstrated that acute infusions of insulin and BCAA improves muscle protein balance in sheep fed at maintenance energy intake but the length of the current experiment necessitated feeding slightly above maintenance. Animals consumed their entire ration and achieved a metabolisable energy intake that was 29% higher than the metabolisable energy requirement for maintenance. This restricted feed intake resulted in a linear increase in live weight over the three months of the experiment of 0.3 ± 0.04 kg/d, approximately one third of their potential maximum rate of growth

Intravenous infusion of glucose caused an increase in average plasma glucose and insulin concentrations over the last 6 hours of the 5 day infusion period (P < 0.05; Table 1). There was no significant variation between the hourly samples taken over the 6 hours for plasma glucose and insulin (results not shown). The increases in plasma glucose (34%) and insulin (83%) were well within the physiological range. Infusion of EAA alone or in addition to glucose did not significantly alter plasma glucose or insulin concentrations compared to control or glucose infusion, respectively. Plasma IGF-1 was increased (73%) by glucose infusion, not altered by EAA infusion and co-infusion of glucose + EAA elicited an additional increase (106%) in IGF-1 concentration compared with glucose alone (P < 0.05; Table 1). There were no significant effects of infusions on mean plasma growth hormone (Table 1) but there was evidence of increased variation in plasma GH concentration over the sampling period during the glucose and glucose + EAA infusions suggesting increased pulsatility (results no shown).

**Table 1:** Mean plasma metabolites and hormones concentrations in calves over the final 6 hours of a five day intravenous infusion of saline (control); glucose; essential amino acids (EAA) and glucose + EAA.

	**Control**	**Glucose**	**EAA**	**EAA+Glucose**
**Glucose mmol/L**	4.1^a ^± 0.20	5.5^bc ^± 0.17	4.4^ac ^± 0.14	5.3^c ^± 0.20
**Insulin ng/ml**	0.6^a ^± 0.03	1.1^b ^± 0.10	0.6^a ^± 0.05	0.9^a ^± 0.10
**IGF – 1 ng/ml**	38.8^a ^± 5.5	67.2^c ^± 6.03	47.4^ac ^± 3.93	80.0^b ^± 6.55
**GH ng/ml**	5.4^a ^± 0.81	5.8^a ^± 0.50	4.7^a ^± 0.50	5.3^a ^± 0.34

Values are averages of 6 blood samples taken at hourly intervals except for GH where samples were collected every 15 min during the last 6 hours of the infusion. Values are means ± standard error of the mean (SEM). ^a,b,c ^Values within the same row with different superscripts are significantly different (P < 0.05)

Infusion of glucose depressed the average concentrations of amino acids in pooled plasma taken from hourly samples. This effect was significant for EAA, and when just BCAA were considered, whether glucose was infused with or without EAA (P < 0.05; Table 2). Analyses of individual amino acids showed that the infusion effects on plasma BCAA concentrations were due to parallel changes in individual BCAA (leucine, isoleucine and valine). Plasma concentrations of, the essential amino acids, phenylalanine, methionine, lysine, histidine, arginine and threonine were significantly increased during the EAA infusion (P < 0.05). Glucose infusion increased the plasma concentrations of some non-essential amino acids (serine, aspartate, glycine), and decreased proline and the urea cycle intermediates, citrulline and ornithine (P < 0.05; Table 2). EAA infusion decreased circulating levels of serine, aspartate, proline and glycine while increasing ornithine.

**Table 2:** Mean plasma amino acids concentrations (μmol/L) in calves on the final day of a five day intravenous infusion of saline (control); glucose; essential amino acids (EAA) and glucose + EAA.

	**Control**	**Glucose**	**EAA**	**EAA+Glucose**
**Leucine**	135^a ^± 7.83	69.8^c ^± 5.22	239.6^b ^± 13.15	155^a ^± 12.05
**Isoleucine**	110^a ^± 6.04	59.1^c ^± 4.09	193.6^b ^± 7.09	131^a ^± 8.77
**Valine**	252^a ^± 14.31	135^c ^± 11.39	465.9^b ^± 24.50	298^d ^± 19.09
**Histidine**	50.5^a ^± 1.96	30.8^c ^± 2.79	70.3^b ^± 1.50	68.6^b ^± 2.93
**Phenylalanine**	60.9^a ^± 2.60	49.0^a ^± 2.62	186.4^b ^± 6.78	185^b ^± 9.80
**Tyrosine**	50.9^a ^± 2.60	43.6^a ^± 4.15	67.5^b ^± 7.04	86.1^b ^± 6.81
**Methionine**	15.8^a ^± 1.31	11.4^a ^± 1.10	34.7^b ^± 2.17	36.9^b ^± 2.03
**Arginine**	129^a ^± 8.26	78.6^c ^± 6.99	169.9^b ^± 3.39	166^b ^± 7.54
**Lysine**	118^a ^± 7.87	56.9^c ^± 5.18	186.1^b ^± 8.84	166^b ^± 10.5
**Threonine**	111^a ^± 7.68	80.2^a ^± 7.61	179.7^b ^± 11.49	202^b ^± 11.24
**Tryptophan**	34.9^ac ^± 2.89	27.7^a ^± 1.87	41.7^bc ^± 1.07	44.7^bc ^± 1.68
**Serine**	76.4 ± 6.15	101 ± 7.29	62.8 ± 4.26	80.7 ± 4.33
**Aspartate**	25.7 ± 0.89	28.1 ± 0.38	18.8 ± 1.42	21.6 ± 0.98
**Asparagine**	29.0 ± 2.26	26.1 ± 1.64	22.7 ± 1.65	25.5 ± 1.06
**Glutamate**	137 ± 12.71	110 ± 5.47	118 ± 9.23	131 ± 8.17
**Glutamine**	188 ± 13.45	197 ± 10.13	154 ± 9.50	164 ± 9.04
**Proline**	77.5 ± 3.90	63.0 ± 3.02	63.1 ± 2.99	63.4 ± 2.02
**Glycine**	221 ± 27.55	386 ± 23.55	156 ± 23.12	292 ± 26.17
**Alanine**	228 ± 5.79	142 ± 9.72	171 ± 11.77	159 ± 8.40
**Citrulline**	73.4 ± 5.28	52.6 ± 3.19	77.6 ± 9.05	66.2 ± 3.54
**Ornithine**	101 ± 8.03	52.2 ± 4.16	117 ± 9.05	84.7 ± 5.71

**TAA**	2272^abc ^± 80.10	1842^ab ^± 106.30	2837^c ^± 74.53	2687^bc ^± 117.92
**BCAA**	497^a ^± 2.95	264^b ^± 27.06	899^c ^± 41.74	584^ac ^± 33.92
**EAA**	1018^a ^± 39.72	598^b ^± 57.86	1767^c ^± 37.42	1336^d ^± 78.87
**NEAA**	1259 ± 52.10	1243^a ^± 7.99	1086^a ^± 48.99	1233^a ^± 7.99

6 plasma samples were taken at hourly intervals during the last 6 hours of the infusion and pooled prior to analysis. TAA, total amino acids; BCAA, branched chain aminoacids; NEAA, non-essential amino acids. Values are means ± standard error of the mean (SEM). ^a,b,c ^Values within the same row with different superscripts are significantly different (P < 0.05).

Plasma urea nitrogen concentrations were significantly (P < 0.05 Table 3) decreased after glucose infusion and were increased (P < 0.05; Table 3) following EAA infusions. Glucose infusion decreased urinary nitrogen excretion and this effect was also observed when glucose was co-infused with EAA, (P < 0.05; Table 3). Urinary nitrogen excretion was increased during EAA infusion such that approximately 50% of the infused EAA nitrogen was excreted. There was a further decrease in urinary nitrogen excretion when glucose and EAA were co-infused with the result that excretion of infused EAA nitrogen was reduced to zero (Table 3). A significant correlation between plasma urea and urinary nitrogen excretion was observed (P < 0.001; results not shown).

**Table 3:** Mean plasma concentration and urinary excretion of urea nitrogen and 3-methylhistidine (3-MH) in calves on the final day of a five day intravenous infusion of saline (control); glucose; essential amino acids (EAA) and glucose + EAA.

	**Control**	**Glucose**	**EAA**	**EAA+Glucose**
**Plasma Urea N mmol/L**	8.8^a ^± 0.91	5.8^b ^± 0.37	11.0^c ^± 0.79	6.7^ab ^± 0.51
**Plasma 3-MH μmol/L**	3.3^a ^± 0.36	1.6^b ^± 0.24	1.9^b ^± 0.61	1.9^b ^± 0.13
**Urinary Urea N g/d**	16.8^a ^± 1.06	12.4^b ^± 1.19	25.3^c ^± 3.70	15.8^ab ^± 1.71
**Urinary 3-MH:Creatinine mg/g**	13.3^a ^± 0.51	12.2^b ^± 0.33	12.5^a ^± 0.24	12.2^a ^± 0.22
**Net Nitrogen Balance g/kg/d**	0.52	0.80	1.06	1.66

Values are averages of 6 blood samples taken at hourly intervals during the last 6 hours of the infusion or pooled urine collected over the last 4 days of infusion. Net nitrogen balance is the difference between digested nitrogen intake (assuming a nitrogen digestibility of 0.85) and urinary nitrogen excretion. Values are means ± standard error of the mean (SEM). ^a,b,c ^Values within the same row with different superscripts are significantly different (P < 0.05).

Plasma 3-MH concentration was significantly (P < 0.05) decreased during infusions of glucose and EAA (Table 3). Urinary 3-MH excretion, was corrected for animal differences in muscle mass by expressing relative to creatinine excretion (3-MH:creatinine), to provide an estimation of skeletal muscle proteolysis. This ratio was significantly decreased after glucose infusion (P < 0.05; Table 3) but co-infusion of EAA did not alter the extent of the inhibitory effect of glucose. Creatinine excretion was not significantly altered by any of the treatments (results not shown). A linear relationship between plasma and urinary 3-MH concentrations was also observed (P < 0.001; results not shown). Estimation of net nitrogen balance assuming a digestible N intake of 25 gN/day and including infused amino N, gave values (g/kg/day) of 0.522 (control); 0.80 (glucose); 1.06 (EAA); 1.66 (glucose + EAA). Therefore co-infusion of glucose and EAA improved nitrogen balance more effectively than individual nutrient infusions.

**Figure 1:**
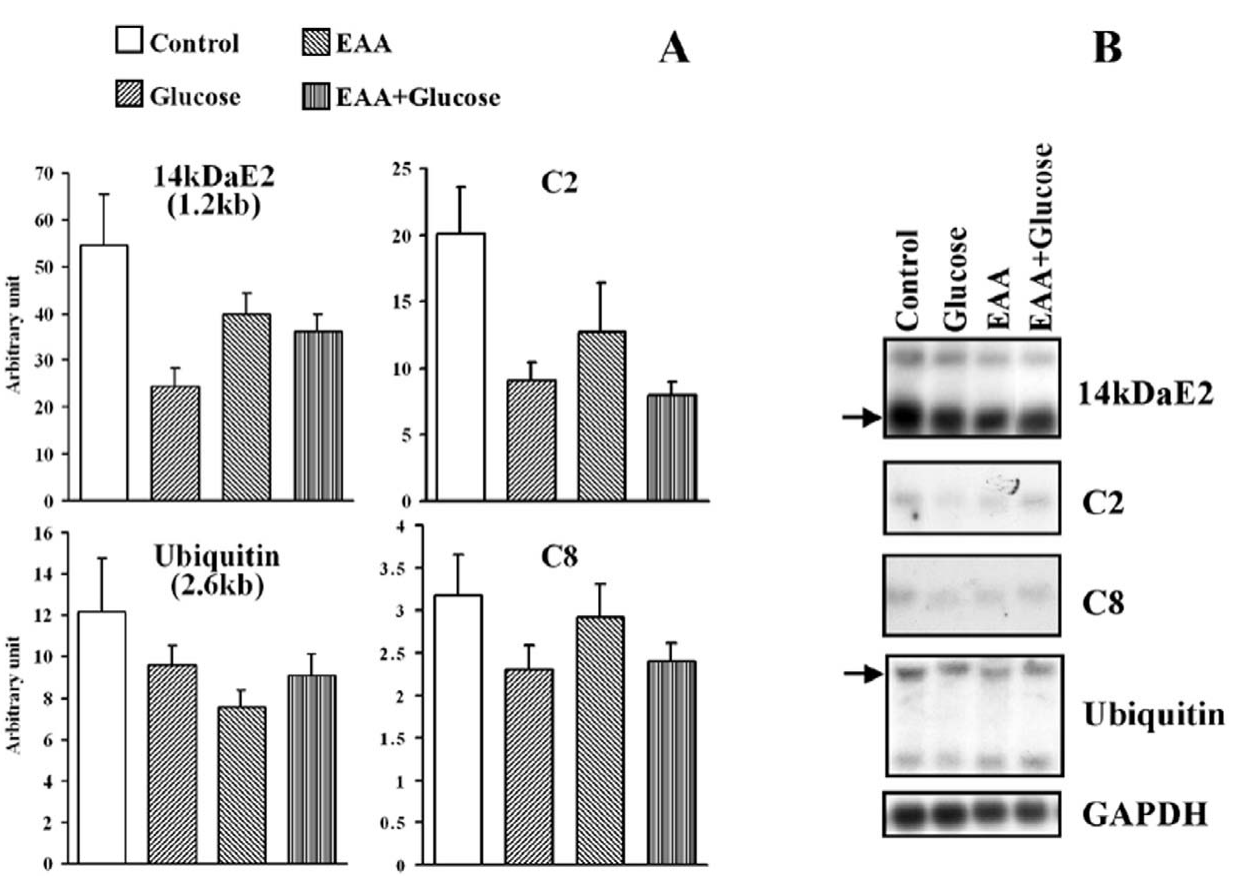
**Expression of the genes encoding components of the ubiquitin proteasome proteolytic pathway**. Muscle biopsies were taken from calves on the final day of a five day intravenous infusion of saline (control); glucose and essential amino acids (EAA). 10 μg of muscle RNA was blotted on a nylon membrane and hybridised against cDNA probes for 14-kDa E2, polyubiquitin, C2 and C8 as described under experimental procedures. A; values are means, normalised to GAPDH, +/- SEM, and represented as arbitrary units. B; representative northern blots.

Expression of ubiquitin conjugating enzyme, 14-kDa E2, and the proteasome sub-unit C2, in skeletal muscle (Figure 1) were significantly reduced after glucose infusion, but not EAA, compared to the control (P < 0.05). There were no significant effects of infusions on muscle mRNA levels of polyubiquitin or the proteasome sub-unit C8 (Figure 1). There was no evidence of synergism between glucose and insulin on expression of components of the ubiquitin-proteasome pathway.

## Discussion

Our study showed that a 5 day intravenous infusion of glucose, sufficient to achieve physiological increases in plasma glucose, insulin and IGF-1 concentrations, improves whole body nitrogen balance, inhibits myofibrillar protein degradation and decreases the expression of components of the ubiquitin proteasome pathway in muscle of normal calves. Infusion of EAA also improved net nitrogen balance but without significant changes in plasma insulin, IGF-1, growth hormone concentrations or muscle protein degradation. We demonstrated a synergistic effect between co-infused glucose and EAA which prevented all of the infused EAA nitrogen from being excreted in urine, but this synergism between glucose and EAA is not due to effects on muscle protein degradation.

Several studies have examined the effects of glucose and amino acid intravenous infusions on nitrogen retention in animals and humans [24-26]. The advantage of supplying glucose is that it is insulinogenic without causing hypoglycaemia, it suppresses non-essential amino acid utilisation for gluconeogenesis and it supplies energy [26]. We report the first study which compares the effects of glucose with infusion of a physiological mixture of only EAA, thereby targeting amino acids to retention in body tissues without the need for deamination of non-essential amino acids. We also report effects of chronic 5 day infusions on myofibrillar protein degradation since parenteral infusions are normally given over extended periods of several days. Previous studies have examined acute nutrient infusions and may give misleading results as several studies have reported transient responses in protein turnover to amino acid infusion [25,27,28].

Glucose infusion alone caused a significant drop in urea synthesis as observed in the decreases in plasma urea levels and urinary urea nitrogen excretion. When the equivalent of 17 gN/day was infused as EAA, 8.5 gN/day or 50% was excreted in the urine, the remaining being retained in body tissues. When glucose was co-infused with EAA, urea nitrogen excretion and plasma urea levels decreased below the control values indicating that all the infused EAA were retained. Estimation of net nitrogen balance demonstrated that glucose infusion alone increased N balance by 4.4 compared with 9.5 gN/day when glucose and EAA were co-infused. Previous work has demonstrated that glucose combined with amino acids given to preoperative patients is the most effective at improving nitrogen balance although these effects occurred without alterations in myofibrillar protein degradation [6,25,26,29,30].

Most studies on the mechanisms by which insulin and amino acids improve protein balance have examined the effects on muscle protein synthesis rather than degradation. Although insulin has been identified as a stimulator of protein synthesis [31], several studies employing amino acid flux determinations in humans, have demonstrated that short-term (up to 3 h) supraphysiological administration of insulin inhibits both whole body protein breakdown and skeletal muscle breakdown [32,10,36]. We measured plasma 3-MH concentration and urinary 3-MH excretion to assess the degradation of myofibrillar proteins in muscle and demonstrated that the increase in nitrogen balance during glucose infusion was accompanied by a significant decrease in muscle protein degradation.

*In vitro *experiments have shown that insulin down regulates proteasome-dependent proteolysis [8,13,37]. The results of our studies here are in agreement with this proposal and demonstrate for the first time that a prolonged 5 day glucose infusion decreases the muscle expression of components of the ubiquitin proteasome pathway. In response to glucose infusion, we observed significant decreases in ubiquitin-conjugating enzyme 14-kDa E2 mRNA and C2 proteasome sub unit, and a small trend for a decrease in Ubiquitin and the C8 proteasome sub unit. Both the ubiquitin-conjugating enzyme E2 and ubiquitin-protein ligase E3 have been suggested to regulate the rate of protein substrate degradation by the proteasome complex [38] while expression of ubiquitin and 26S proteasome subunits are thought to adapt to changes in flux through the pathway rather than being regulatory.

Despite the additive effect between co-infused glucose and EAA on nitrogen excretion and nitrogen balance, these treatments did not further decrease myofibrillar protein degradation values or the expression of components of the ubiquitin proteasome pathway, observed with glucose only infusion. Acute infusion experiments *in vivo *in humans and rodents [9,33], have provided conflicting evidence for a synergistic effect of amino acids on insulin suppression of muscle proteolysis. It has been suggested that proteasome-dependent muscle proteolysis may only respond over a longer time period (10 h) to nutrients [9] but this was not apparent in our study. These *in vivo *studies contrast with our C2 C12 *in vitro *experiments which show that amino acids enhance the inhibitory effects of insulin on proteolysis and that this effect involves both the suppression of ubiquitin-dependent proteolysis and is associated with reduced E2 expression [8].

Acute (3–5 h) amino acid infusion in to humans has been reported to decrease protein breakdown in skeletal muscle and splanchnic tissue [39,40] but other have failed to confirm these findings [41,42]. Volpi *et al*. (1998) [43] showed that amino acid infusion did not effect the degradation of myofibrillar proteins, measured by 3-methylhistidine release in elderly humans under conditions where they did not observe any change in plasma concentration of insulin and IGF-1.

Studies in healthy humans have demonstrated the stimulatory effect of infused amino acids or high dietary protein intake on skeletal muscle protein synthesis [43,44]. The synergistic effect of glucose and EAA in nitrogen retention may therefore be due to stimulation of muscle protein synthesis. In rodents, acute BCAA infusions stimulate the sensitivity of muscle protein synthesis to the anabolic effects of co-infused insulin [7]. Our in vivo studies in maintenance-fed sheep [14] failed to confirm these findings while Bohe *et al*. (2003) [28] have demonstrated that the response of human muscle protein synthesis to increased availability of amino acids in plasma, is transient.

Co-infusion of glucose and EAA may also have altered protein turnover in tissues other than muscle since Nygren and Nair, (2003) [40], showed that amino acids have an inhibitory effect on splanchnic protein breakdown which was independent of insulin. Suppression of proteolysis observed *in vivo *may also be mediated through the autophagic pathway since in liver and visceral tissues, this is the major proteolytic pathway, and is sensitive to amino acid regulation, independent of insulin [45].

The additive effect of glucose and EAA on nitrogen retention was accompanied by a similar effect on plasma IGF-1 concentrations. Our previous studies in sheep have demonstrated that that prolonged (5 day) glucose infusion is able to induce an IGF-1 secretory response to GH administration only when dietary protein intake is increased [46]. Several studies in patients undergoing surgery have demonstrated that increased IGF-1 levels are associated with improved nitrogen balance [47]. Furthemore improvements in postoperative N balance due to AA infusion have been associated with increased IGF-1 in plasma [48]. These responses may be due to changes in muscle protein synthesis, since IGF-1 administration to rats does not inhibit muscle proteolysis [49])

## Conclusion

We conclude that chronic 5-day intravenous infusions of glucose and EAA in calves act synergistically to improve whole body nitrogen balance but although glucose infusion decreased myofibrillar protein degradation and muscle expression of components of the ubiquitin proteasome pathway, the synergism between glucose and EAA is not due to effects on muscle protein degradation.

## Abbreviations

BCAA, branched chain amino acid; EAA, essential amino acid; GAPDH, glyceraldehyde 3 phosphate dehydrogenase; IGF-1, insulin like growth factor 1; GH, growth hormone; HPLC, high performance liquid chromatography; 3-MH, 3-methylhistidine.

## Competing interests

The author(s) declare that they have no competing interests.

## Authors' contributions

MAL, FS, JRS and LAC conceived the study; FS and MAL drafted the manuscript; All authors have approved the final manuscript.
